# Environmental risk factors for *Toxoplasma gondii* infections and the impact of latent infections on allostatic load in residents of Central North Carolina

**DOI:** 10.1186/s12879-018-3343-y

**Published:** 2018-08-23

**Authors:** Andrey I. Egorov, Reagan Converse, Shannon M. Griffin, Jennifer Styles, Elizabeth Klein, Elizabeth Sams, Edward Hudgens, Timothy J. Wade

**Affiliations:** 1 United States Environmental Protection Agency, National Health and Environmental Effects Research Laboratory, MD 58-C, 109. T.W. Alexander Drive, Research Triangle Park, NC 27711 USA; 2 United States Environmental Protection Agency, National Exposure Research Laboratory, Cincinnati, OH USA; 3 Gillings School of Global Public Health, Environmental Sciences and Engineering Department, University of North Carolina at Chapel Hill, Chapel Hill, NC USA; 4 ORAU Student Services Contractor to US EPA, Chapel Hill, NC USA

**Keywords:** *Toxoplasma gondii*, Seroprevalence, Vegetated land cover, Biomarkers, Allostatic load

## Abstract

**Background:**

*Toxoplasma gondii* infection can be acquired through ingestion of infectious tissue cysts in undercooked meat or environmental oocysts excreted by cats. This cross-sectional study assessed environmental risk factors for *T. gondii* infections and an association between latent infections and a measure of physiologic dysregulation known as allostatic load.

**Methods:**

Serum samples from 206 adults in the Durham-Chapel Hill, North Carolina area were tested for immunoglobulin (IgG) responses to *T. gondii* using commercial ELISA kits. Allostatic load was estimated as a sum of 15 serum biomarkers of metabolic, neuroendocrine and immune functions dichotomized at distribution-based cutoffs. Vegetated land cover within 500 m of residences was estimated using 1 m resolution data from US EPA’s EnviroAtlas.

**Results:**

Handling soil with bare hands at least weekly and currently owning a cat were associated with 5.3 (95% confidence limits 1.4; 20.7) and 10.0 (2.0; 50.6) adjusted odds ratios (aOR) of *T. gondii* seropositivity, respectively. There was also a significant positive interaction effect of handling soil and owning cats on seropositivity. An interquartile range increase in weighted mean vegetated land cover within 500 m of residence was associated with 3.7 (1.5; 9.1) aOR of *T. gondii* seropositivity. Greater age and consumption of undercooked pork were other significant predictors of seropositivity. In turn, *T. gondii* seropositivity was associated with 61% (13%; 130%) greater adjusted mean allostatic load compared to seronegative individuals. In contrast, greater vegetated land cover around residence was associated with significantly reduced allostatic load in both seronegative (*p* < 0.0001) and seropositive (*p* = 0.004) individuals.

**Conclusions:**

Residents of greener areas may be at a higher risk of acquiring *T. gondii* infections through inadvertent ingestion of soil contaminated with cat feces. *T. gondii* infections may partially offset health benefits of exposure to the natural living environment.

## Background

*Toxoplasma gondii* is a ubiquitous protozoan parasite that infects felines as definitive hosts and a wide range of other warm-blooded animals, including humans, as intermediate hosts. Infected cats excrete copious amounts of *T. gondii* oocysts containing infectious sporozoites in their feces. The oocysts can contaminate soils and remain viable for months to years [1, 2]; subsequent ingestion of oocyst-contaminated soil causes infection in intermediate hosts. *T. gondii* infects many tissues of intermediate hosts, including muscles and the central nervous system, where it forms infectious tissue cysts. Predation of intermediate hosts by felines completes the life cycle of the parasite. In humans, life-long infections usually result from ingestion of raw or undercooked meat of infected intermediate hosts, such as pigs, as well as ingestion of environmental oocysts [3]. Other infection routes in humans include vertical transmission from infected mother to her fetus and transmission via blood transfusion or organ transplant [4, 5]. A new infection typically involves a transient acute phase caused by the rapidly replicating tachyzoites of the parasite followed by a latent, life-long stage with bradyzoites persisting inside tissue cysts.

Serum immunoglobulin (Ig) M response to the parasite is characteristic of the acute phase of infection. Serum IgG response, which reaches a maximum level within 2 to 3 months of initial infection and then slowly declines to a residual elevated level, is characteristic of the latent phase of infection [4]. A combination of serum IgG and IgM responses to the parasite is used to differentiate acute and latent infection phases in diagnostic settings [6]. Serum IgG immunoassays are a standard test used in population surveillance of *T. gondii* seroprevalence [7, 8].

According to the nationally representative data from the National Health and Nutrition Examination Survey (NHANES), IgG seroprevalence of *T. gondii* in the US in individuals older than 5 years of age was 13.2% in 2009–2010. There had been a substantial decline in seroprevalence during the previous 20-year interval [8]. Low socioeconomic status has been linked with increased odds of seropositivity in the US [9].

There is strong epidemiologic evidence of *T. gondii* transmission via consumption of raw or undercooked meat [10, 11] suggesting that ingestion of tissue cysts is a dominant infection pathway in the US and other developed countries. Research using antibody responses to sporozoite-specific antigen demonstrated that ingestion of environmental oocysts containing sporozoites is also a common infection pathway in North America [12, 13]. Some studies also provided evidence of an association between contacts with cats and *T. gondii* infection in developed countries [10, 11, 14], while other studies failed to confirm this association [15–17]. Associations between gardening and other soil contacts with risk of *T. gondii* infection have also been demonstrated [15, 16, 18]. In addition, waterborne outbreaks of toxoplasmosis have been reported in Canada [19] and Brazil [20].

Only 10–30% of new *T. gondii* infections in humans cause clinical symptoms [21], but when symptoms are present, the clinical manifestations of the disease can be severe. Symptoms of acute toxoplasmosis include ocular disease, encephalitis, chorioretinitis, lymphadenitis or lymphadenopathy, and myocarditis [22]. *T. gondii* infection during pregnancy and vertical transmission of the parasite to the fetus can cause mental disabilities, seizures, blindness and spontaneous abortion [3]. In the US, 400 to 4000 infants are born annually with congenital toxoplasmosis [3, 23].

While the latent phase of infection may appear asymptomatic, there is a growing body of evidence of behavioral modifications in intermediate hosts enhancing the probability of predation by felines. Examples from animal studies include fatal attraction to and sexual arousal by the smell of cat urine in infected rats [24, 25], and similar fatal attraction to the smell of leopard urine in infected chimpanzees [26]. Epidemiological studies have also linked latent infections in humans with adverse neuropsychological outcomes, including elevated risk of suicide [27], impaired reaction time and increased risk of traffic accidents [28], and mental health disorders including schizophrenia, depression, and obsessive compulsive disorder [22], as well as greater odds of developing a metabolic disorder type 2 diabetes [29]. There is also evidence of widespread immune activation and subclinical neurophysiological changes induced by *T. gondii* infection in humans [30, 31]. However, the knowledge of sub-clinical health effects of latent infections and biological pathways leading to these effects remains rather limited.

The objectives of this study were: (i) to assess behavioral and environmental risk factors for *T. gondii* infections in the Durham-Chapel Hill, NC area, and (ii) to explore potential associations between latent *T. gondii* infections and a composite biomarker-based measure of physiological dysregulation known as allostatic load (AL), and individual biomarkers of immune, neuroendocrine and metabolic functions.

## Methods

### Study design and data collection

The protocol of this cross-sectional population-based observational study was approved by the Institutional Review Board of the University of North Carolina at Chapel Hill. The target population included adult (at least 18 years of age) residents of the Durham-Chapel Hill metropolitan area in North Carolina. The study was advertised on the US EPA’s website for recruitment of volunteers in epidemiological research, and by displaying study posters at various venues. Veterinary practices and animal shelters were targeted for recruitment to over-sample individuals with increased contacts with cats, dogs and other animals. Participants reported to the US EPA Human Studies Facility (HSF) in Chapel Hill, NC. A venous blood sample was drawn in a BD Vacutainer SST tube (Becton, Dickinson and Company, Franklin Lakes, NJ), and height and weight were measured by a registered nurse. Serum was separated following manufacturer instructions on the day of collection, and stored at − 80° C until analysis. Participants also completed a questionnaire addressing their demographic and socioeconomic characteristics, and behavioral and environmental factors which may be associated with exposure to *T. gondii*, such as contacts with cats, handling soil and consumption of undercooked meat of various types. Data collection was conducted in May – September 2013.

### Serological tests

Serum samples were tested for IgG response to *T. gondii* using VIR-ELISA Anti-Toxo IgG assays (VIRO-IMMUN Labor-Diagnostika GmbH, Oberursel, Germany) in accordance with manufacturer’s instructions. Geometric mean values from duplicate tests were used in data analysis. Samples from two individuals with indeterminate results (average optical density values within plus/minus 10% interval around the plate-specific cut-off) were re-analyzed. If a new test again produced an indeterminate result, the infection status was classified as negative if the average ratio of optical density value for the sample to the corresponding plate-specific cut-off was less than one and as positive otherwise.

As part of a previously conducted study of environmental predictors of AL [32], serum samples were also analyzed for 15 stress-related biomarkers, including nine biomarkers of immune function: C-reactive protein (CRP), vascular cell adhesion molecule 1 (VCAM-1), intercellular adhesion molecule 1 (ICAM-1), interleukin (IL)-1β, IL-6, IL-8, tumor necrosis factor (TNF)-α, fibrinogen, and myeloperoxidase (MPO); four biomarkers of neuroendocrine function: dehydroepiandrosterone (DHEA), epinephrine, norepinephrine, and dopamine; and two biomarkers of metabolic function: uric acid and serum amyloid A (SAA). All biomarker tests were conducted using commercially available assay kits as described previously [32].

### Analysis of allostatic load

AL was calculated as a sum of dichotomized biomarker values, which is the most commonly used approach in AL studies [33]. Biomarker data were dichotomized at the 10th percentile of the sample distribution (DHEA and dopamine), at the 90th percentile (IL-1β, IL-6, IL-8, TNF-α, fibrinogen, uric acid, MPO, CRP, SAA, VCAM-1, and ICAM-1) or at both 10th and 90th percentiles (norepinephrine and epinephrine, two binary variables for each biomarker), depending on which tail of the biomarker distribution is known to be associated with an elevated risk of disease or death. Thus, AL measures were based on a total of 17 binary variables representing 15 biomarkers as described previously [32].

### Analysis of residential exposure to living environment

Proportions of total vegetated land cover within a 500 m radius of each residence were estimated using high resolution land cover data for the Durham-Chapel Hill, NC metropolitan area from the US EPA’s mapping application EnviroAtlas (https://www.epa.gov/enviroatlas) as described previously [32]. Vegetated land cover was defined as the sum of two land cover categories: Trees & forest, and Grass & other herbaceous. Exposure measures were based on average proportion of vegetated land cover within 50 m, 150 m, and 500 m radii, and distance-weighted average proportion of vegetated land cover within 500 m radius around the residence. The latter measure was calculated as an arithmetic mean of vegetated land cover proportions for ten concentric 50 m annuli from 0-50 m to 450–500 m. This weighting scheme implicitly used a constant weight of 0.1 for each annulus. As a result, a square meter of vegetated land cover within the 0–50 m annulus (7854 m^2^ area) had 19 times greater impact on the weighted estimate than a square meter of vegetated land cover within the 450–500 m annulus with 19 times larger area (149,226 m^2^).

### Statistical data analysis

Statistical analysis was conducted using SAS version 9.4 software (SAS Institute, Cary, NC). It involved two phases: the first phase focused on environmental predictors of *T. gondii* infections with *T. gondii* serostatus being an outcome variable while the second phase focused on subclinical health outcomes of latent *T. gondii* infections. In the second phase, *T. gondii* serostatus was a predictor variable while AL and individual biomarkers were modeled as outcome variables, one variable at a time.

At the first phase, univariate analysis of associations between demographic, behavioral and environmental factors and *T. gondii* infections was conducted using the Chi-square Wald test for binary and nominal variables and the Cochran-Armitage test for trend for ordinal variables. Subsequent multivariate regression analysis involved developing predictive models of *T. gondii* seropositivity. An initial predictive logistic regression model included a set of socio-demographic and behavioral covariates. The next step involved adding cat-related variables to the initial model, one variable at a time, and selecting the cat variable that produced the best model fit. Akaike Information Criterion Corrected (AICc) values in the output of SAS procedure *genmod* were used as a measure of model fit.

The next step involved adding vegetated land cover measures to the previously developed model, one variable at a time. To account for spatial autocorrelation, all regression models for vegetated land cover included a two-dimensional spline smoothing function of geographic coordinates (“thin-plate smoother”), as described previously [32]. The models involving a combination of linear and non-linear predictors also known as generalized additive models were fitted using the SAS procedure *gam*. Using generalized additive models is a common approach in analysis of geographic distributions of health outcomes [34–36]. The spline function was fitted using the option “method = GCV” (generalized cross-validation function) for automatically selecting degrees of freedom which define the flexibility of the “thin plate” smoother. Model fit was assessed using the deviance of the final estimate criterion from the output of SAS procedure *gam*.

In the second phase, associations of *T. gondii* seropositivity with AL (a Poisson-distributed count variable), as well as individual dichotomized biomarkers and continuous log-transformed biomarkers were analyzed in univariate and in multivariate regression models adjusting for demographic and socioeconomic covariates. The univariate analysis of association between *T. gondii* and AL was followed by developing a multivariate predictive Poisson regression model including demographic covariates and body mass index (BMI). A final predictive model for AL also included vegetated land cover as a covariate and a spline function of geographic coordinates to account for spatial autocorrelation; it was fitted using the SAS procedure *gam* as described above. Associations between *T. gondii* seropositivity and individual biomarkers were analyzed using logistic regression models for binary biomarkers adjusting for socio-demographic covariates or linear multivariate models for log-transformed biomarker data.

## Results

### Predictors of *T. gondii* seropositivity

#### Descriptive statistics, univariate analysis and associations among covariates

Two-hundred and six individuals residing at 173 street addresses were included in the present study. Among them, there were 17 (8.3%) *T. gondii* seropositive individuals (Table 1). The mean age of participants was 37.4 years with a range from 18 to 85 years. As expected, *T. gondii* seropositivity was associated with increased age (*p* < 0.0001) in univariate analysis. Sex, race and ethnicity status (dichotomized as non-Hispanic Whites (53.7%) vs. all others), and education (dichotomized as 4-year college degree or greater (51.5%) vs. less than college degree) were not associated with seropositivity. Obesity (28.6% of participants) was significantly associated with seropositivity in a univariate model. Approximately half of the study participants (51.5%) lived in Durham (city with 258,000 residents in 2015), 29.1% lived in Chapel Hill (city with a large university campus; 59,000 residents), and the remaining 19.4% lived in other towns in the Durham-Chapel Hill metropolitan area. Seroprevalence rates did not differ significantly among these locations. Individuals who handled soil with bare hands at least one time per week were significantly more likely to be seropositive in the univariate analysis. While current cat ownership was a significant risk factor, past cat ownership was not associated with seropositivity. Other variables significantly associated with *T. gondii* in univariate analysis included having outdoor cats, and a total duration of cat ownership of 4 years or longer.

**Table 1:** Descriptive statistics of the study population and univariate analysis of risk factors for *T. gondii* seropositivity

Variable	Level	Participants, N (%)	*T. gondii* seropositive, N (%)	*p* value
All participants		206 (100%)	17 (8.3%)	
Age	18–30	88 (42.7%)	2 (2.3%)	
	31–45	62 (30.1%)	5 (8.1%)	
	46–85	56 (27.2%)	10 (17.9%)	0.001^a^
Sex	Males	70 (34.0%)	5 (7.1%)	
	Females	136 (66.0%)	12 (8.8%)	0.7
Race and ethnicity	White non-Hispanic	110 (53.4%)	8 (7.3%)	
	All others	95 (46.1%)	9 (9.5%)	0.6
Education	Less than bachelor’s degree	100 (48.5%)	10 (10.0%)	
	Bachelor’s degree or higher	106 (51.5%)	7 (6.6%)	0.4
Obesity	Not obese	147 (71.4%)	8 (5.4%)	
	Obese	59 (28.6%)	9 (15.3%)	0.03
Smoker	No	148 (71.8%)	9 (6.1%)	
	Yes	57 (27.7%)	8 (14.0%)	0.07
Residence	Chapel Hill	60 (29.1%)	4 (6.7%)	
	Durham	106 (51.5%)	10 (9.4%)	
	Other towns	40 (19.4%)	3 (7.5%)	0.8
Drinking water source at home	Private well or bottled water	67 (32.5%)	5 (7.5%)	
	Municipal system	136 (66.0%)	12 (8.8%)	0.7
Housing unit density	< 4 per acre	178 (86.4%)	14 (7.9%)	
	> = 4 per acre	28 (13.6%)	3 (10.7%)	0.6
Ever lived on a farm	No	169 (82.0%)	11 (6.5%)	
	Yes	37 (18.0%)	6 (16.2%)	0.06
Eat raw/undercooked chicken	No	187 (90.8%)	13 (7.0%)	
	Yes	14 (6.8%)	3 (21.4%)	0.07
Eat raw/undercooked pork	No	188 (91.3%)	14 (7.4%)	
	Yes	12 (5.8%)	2 (16.7%)	0.27
Eat raw/undercooked beef	No	127 (61.7%)	12 (9.4%)	
	Yes	74 (35.9%)	4 (5.4%)	0.31
Handles soil with bare hands weekly	No	151 (73.3%)	8 (5.3%)	
	Yes	53 (25.7%)	8 (15.1%)	0.03
Currently has at least one dog	No	62 (30.1%)	8 (12.9%)	
	Yes	144 (69.9%)	9 (6.3%)	0.12
Currently has at least one cat	No	115 (55.8%)	5 (4.3%)	
	Yes	91 (44.2%)	12 (13.2%)	0.03
Current outdoor cats	Do not have cats	115 (55.8%)	5 (4.3%)	
	Has cats that don’t go outdoors	30 (14.6%)	2 (6.7%)	
	Has cats, let them go outdoors	61 (29.6%)	10 (16.4%)	0.007^a^
Total years lived with cats	0–3 years	115 (55.8%)	4 (3.5%)	
	4 years or more	91 (44.2%)	13 (14.3%)	0.01
Ever been responsible for cleaning a cat litter box	No	75 (36.4%)	3 (4.0%)	
	Yes	130 (63.1%)	13 (10.0%)	0.14

^a^Cochran-Armitage test for trend was used; for all other variables, Chi-square Wald test was used

Stratified univariate analysis demonstrated that regularly handling soil with bare hands was associated with higher odds of *T. gondii* seropositivity only among current cat owners who allow their cats to go outdoors (Table 2). Among five *T. gondii* seropositive individuals who did not have outdoor cats and did not handle soil with bare hands regularly, none (0%) were non-Hispanic White, four (80%) were obese, and only one (20%) attained at least bachelor’s level education, while the average age in this group was 58.6 years. In contrast, among seven seropositive individuals who had outdoor cats and handled soil regularly, six (86%) were non-Hispanic White, two (29%) were obese, and four (57%) attained a bachelor’s level education, while the average age in this group was 39.7 years.

**Table 2:** Stratified descriptive statistics on *T. gondii* seropositivity

Strata	Substrata	Participants	*T. gondii* seropositive	*p* value
No outdoor cats	Does not handle soil weekly	112 (54.4%)	5 (4.5%)	
	Handles soil with bare hands weekly	31 (15.0%)	1 (3.2%)	0.76
Outdoor cats present	Does not handle soil weekly	39 (18.9%)	3 (7.7%)	
	Handles soil with bare hands weekly	22 (10.7%)	7 (31.8%)	0.02

The median proportions of vegetated land cover within 50 m and 500 m around residence were 59.9 and 72.8%, respectively (Table 3); statistics for 150 m radius and for the distance-weighted measure of land cover were between these estimates. Interquartile ranges (IQR) varied from 15.0% for average vegetated land cover within 500 m to 25.9% for the 50 m radius. Average housing unit density at a census block-group level varied from 0.08 to 11.2 (median 1.53, IQR 1.93) housing units per acre or from 0.20 to 27.7 (median 3.78) housing units per hectare.

**Table 3:** Percent of vegetated land cover within specified distance from residence

Measure of vegetated land cover	Median	Min - max	IQR
50 m average	59.9	6.0–99.8	25.9
150 m average	67.6	19.5–94.8	21.7
500 m average	72.8	36.9–95.2	15.0
500 m weighted	68.7	31.7–94.3	17.4

Exploratory analysis of predictors of current cat ownership (dichotomized as at least one cat vs. no cats) demonstrated that current dog ownership (dichotomized similarly) was inversely associated with cat ownership (aOR = 0.10, 95% confidence limits 0.04; 0.24), while non-Hispanic White ethno-racial status was associated with a 16.1 (7.00; 37.0) aOR of current cat ownership compared to non-White and Hispanic individuals. An IQR increase in housing unit density was linked with 0.53 (0.29; 0.98) OR of owning a cat that roamed outdoors. Individuals with greater vegetated land cover around their residence were more likely to handle soil with bare hands at least weekly. The strongest effect was observed for distance-weighted proportion of vegetated land cover within 500 m: an IQR increase in this measure of vegetated land cover was associated with a 1.68 (1.04; 2.71) aOR of handling soil after adjusting for race/ethnicity and spline function of geographic coordinates.

#### Multivariate regression analysis of predictors of *T. gondii* seropositivity

A preliminary predictive model of seropositivity included age, smoking status, obesity, eating undercooked pork, average housing unit density per census block group, and handling soil with bare hands at least weekly. Further analysis of cat-related variables identified currently owning a cat as the strongest predictor of *T. gondii* seropositivity. The resulting multivariate predictive model of seropositivity (Model 1) is presented in Table 4.

**Table 4:** Results of multivariate regression analysis of predictors of *T. gondii* seropositivity; adjusted odds ratios (aOR) with 95% confidence limits. Model 1 – logistic model for socio-demographic and behavioral predictors; Model 2 – logistic model with an interaction effect; Model 3 – generalized additive model including vegetated land cover

Predictor	Model 1	Model 2	Model 3
Age (years)	1.08 (1.03, 1.14)^*^	1.09 (1.03, 1.15)^*^	1.12 (1.06, 1.17)^*^
Current smoking	5.74 (1.29, 25.6)^*^	5.16 (1.11, 23.9)^*^	7.68 (1.78, 33.1)^*^
Obesity	2.76 (0.67, 11.4)	4.67 (1.01, 21.6)^*^	
Eating undercooked pork	5.68 (0.81, 39.9)	4.89 (0.71, 33.7)	10.1 (1.52, 67.7)^*^
Housing unit density, units/acre	1.30 (0.95, 1.77)		1.30 (0.92, 1.83)
Handling soil weekly	5.34 (1.37, 20.7)^*^	0.35 (0.03, 4.19)	
Currently owning a cat	10.0 (1.98, 50.6)^*^		12.1 (2.86, 51.5)^*^
Owning an outdoor cat		1.07 (0.16, 7.36)	
Interaction effect of handling soil and owning outdoor cat		82.2 (2.75, 2454)^*^	
Two-dimensional spline function of geographic coordinates			*p* = 0.07
Vegetated land cover within 500 m weighted, IQR increase			3.67 (1.48, 9.08)^*^

^*^p < 0.05

Further analysis involved assessing an interaction effect of handling soil with bare hands and being exposed to cats. In this analysis, a binary variable for owning a cat that roamed outdoors produced the best model fit (Model 2, Table 4). The interaction effect of handling soil and owning an outdoor cat on odds of seropositivity was statistically significantly positive (*p* = 0.01). The interaction effect of handling soil and owning any cats was also statistically significant with *p* = 0.03 (not shown). Housing unit density was excluded from these models because preliminary analysis demonstrated that it was inversely associated with allowing cats to go outdoors.

Model 3 (Table 4) was developed to assess an association between vegetated land cover and *T. gondii* seropositivity. Preliminary analysis involved fitting four models with measures of average vegetated land cover within 50 m, 150 m, or 500 m of residence, or 500 m distance-weighted average, one measure at a time (Table 5). The distance-weighted average measure produced the best model fit. In this model, an IQR increase (17.4% increase) in vegetated land cover was associated with a 3.67 (1.48; 9.08) aOR of *T. gondii* seropositivity (*p* = 0.006). Obesity was excluded from Model 3 as it was no longer a significant predictor of seropositivity. Handling soil with bare hands was also excluded from Model 3 because preliminary analysis demonstrated that greater vegetated land cover was associated with greater odds of handling soil. An additional analysis demonstrated that adjusting for handling soil with bare hands reduced the effect estimate for vegetated land cover to 2.94 (1.13; 7.68) indicating that more frequent contacts with soil contaminated with *T. gondii* oocysts partially mediated the observed association between residential vegetated land cover and *T. gondii* infections.

**Table 5:** Comparison of associations between vegetated land cover measure and *T. gondii* seropositivity using generalized additive models (covariates as in Model 3 in Table 4)

Vegetated land cover measure	IQR, %	Model deviance	aOR of *T. gondii* seropositivity with 95% confidence limits
50 m average	25.9	68.94	2.39 (1.09, 5.24)^*^
150 m average	21.7	68.37	2.85 (1.20, 6.76)^*^
500 m average	15.0	67.79	3.01 (1.29, 7.00)^*^
500 m weighted average	17.4	67.13	3.67 (1.48, 9.08)^*^

^*^p < 0.05

#### Latent *T. gondii* infection as a predictor of physiologic dysregulation

In this analysis, *T. gondii* serostatus was modelled as a predictor variable while outcome variables were AL or individual biomarkers. AL data were available for a subset of 186 (90.3%) study participants. AL index values varied from 0 to 9 with a median of 1 and a mean of 1.70. Detailed statistics on individual biomarkers are available in the previously published manuscript [32].

In a univariate Poisson regression model, the estimated mean AL was 69% (22%; 135%), *p* = 0.002 greater among *T. gondii* seropositive individuals compared to seronegative controls (Model 4, Table 6). A multivariate model adjusting for age, race/ethnicity, education, and log-transformed BMI also produced a statistically significant association between *T. gondii* infection and AL (Model 5). The full predictive model for AL (Model 6) included, in addition to the above covariates, average distance-weighted vegetated land cover within 500 m radius of residence. The results show that greater vegetated land cover was a highly significant (*p* < 0.0001) negative predictor of AL indicating a beneficial effect of exposure (Table 6). The observed adjusted multiplicative effect on mean AL per IQR increase in vegetated land cover was 0.62 (0.54; 0.72); in other words, the predicted mean AL was 38% (28%; 46%) lower in individuals with greater vegetated land cover around their residences. The detrimental effect of *T. gondii* infection on AL was independent of the effect of green spaces: the mean AL was 61% (13%; 130%) greater in seropositive individuals compared to seronegative controls in Model 6.

**Table 6:** Results of regression analysis of predictors of AL, adjusted multiplicative effects on AL with 95% confidence limits

Predictor variable	Model 4	Model 5	Model 6
Age, year		1.01 (1.001, 1.02)^*^	1.01 (1.004, 1.02)^*^
Non-Hispanic White race/ethnicity		1.20 (0.94, 1.54)	1.37 (1.06, 1.78)^*^
Education (bachelor’s degree or higher)		0.83 (0.65, 1.06)	0.73 (0.57, 0.94)^*^
Log_10_(BMI)		12.7 (4.15, 39.1)^*^	10.8 (3.57, 32.9)^*^
Vegetated land cover, per IQR increase			0.62 (0.54, 0.72)^*^
Two-dimensional spline function of geographic coordinates			*p* = 0.04
*T. gondii* seropositivity	1.69 (1.22, 2.35)^*^	1.43 (1.01, 2.03)^*^	1.63 (1.15, 2.33)^*^

^*^p < 0.05

Additional stratified analysis in subsets of *T. gondii* seropositive (15 individuals with AL data) and seronegative (*N* = 171) study participants using a similar set of covariates demonstrated that exposure to vegetated land cover was statistically inversely associated with AL in both subsets. Multiplicative effect estimates per IQR increase in vegetated land cover were 0.26 (0.14; 0.49), *p* = 0.004 and 0.63 (0.54; 0.73), *p* < 0.0001 in seropositive and seronegative individuals, respectively.

**Fig. 1:**
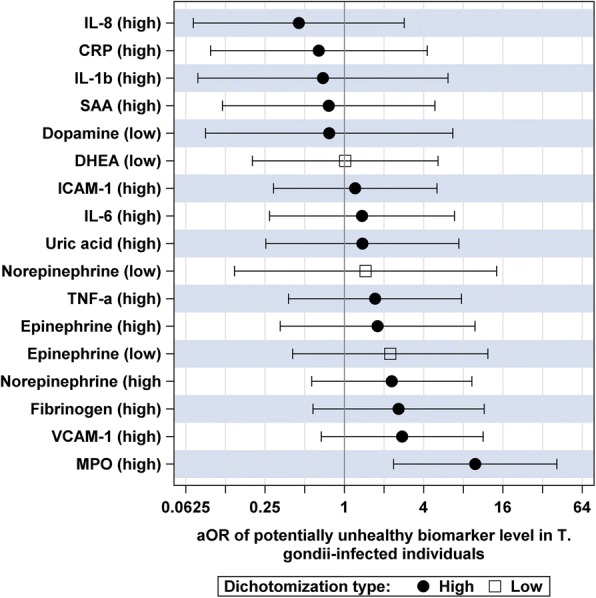
Adjusted odds ratios (aOR) of having potentially unhealthy biomarker levels (high level above the 90th percentile or low level below the 10th percentile depending on the biomarker) in *T. gondii* seropositive individuals vs. seronegative controls

Further analysis focused on assessing effects of latent *T. gondii* infections on dichotomized biomarkers. Regression models included age, sex, race/ethnicity, education, smoking status, and log-transformed BMI as covariates. Adjusted odds ratios of having potentially unhealthy biomarker values either below the 10th percentile or above the 90th percentile of sample distribution are presented in Fig. 1. Serum MPO was the only biomarker significantly associated with *T. gondii* infection with 9.85 (2.36; 41.0), *p* = 0.0017 aOR of biomarker value above the 90th percentile in seropositive individuals compared to seronegative controls. The observed association with MPO remained statistically significant after applying a Bonferroni correction for conducting exploratory analysis of 17 biomarkers, which reduced the cut-off for statistical significance to α* = 0.05/17 =  0.003. The effect estimates for 12 of 17 (71%) biomarkers were greater than one (Fig. 1) suggesting increased odds of having potentially unhealthy biomarker values in *T. gondii* seropositive individuals. The predominance of positive associations with dichotomized biomarkers explains the highly statistically significant effect of *T. gondii* on AL measures comprised of these biomarkers.

**Fig. 2:**
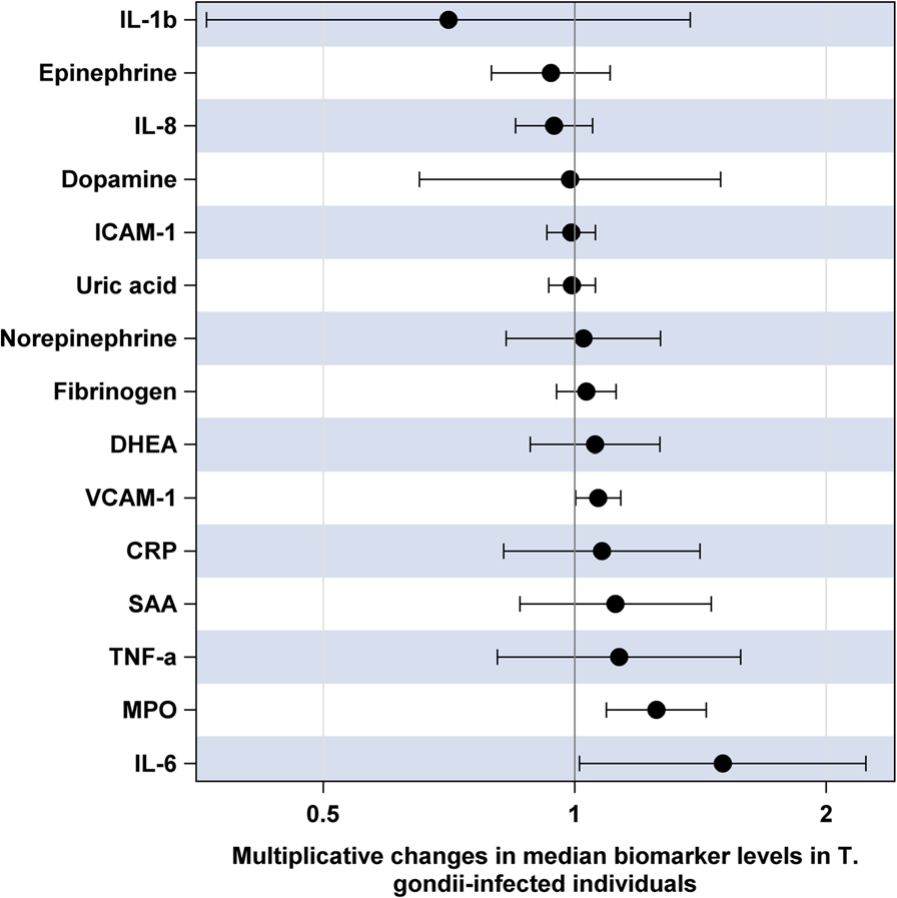
Adjusted multiplicative changes in median biomarker levels in *T. gondii* seropositive individuals compared to seronegative controls

Analysis of associations between *T. gondii* seropositivity and continuous log-transformed biomarkers involved a similar set of covariates. The results (Fig. 2) demonstrated that *T. gondii* seropositivity was associated with 25% (9%; 44%) higher adjusted median concentration of serum MPO (*p* = 0.001), 50% (1%; 123%) higher level of IL-6 (*p* = 0.04) and 7% (0.3%; 13%) higher level of VCAM-1 (*p* = 0.04). Only the association with MPO remained statistically significant after applying a Bonferroni correction for multiple testing.

## Discussion

In this study of urban and suburban adult residents of central North Carolina, greater residential vegetated land cover was significantly associated with *T. gondii* seropositivity. In previous studies, exposures to urban green spaces and other natural living environments have been linked to reduced morbidity and mortality; however, evidence of potential detrimental effects of green spaces including zoonotic infections remained rather sparse [37]. To our knowledge, this is the first study linking residential greenness to *T. gondii* infections.

The present study also found that handling soil with bare hands was a risk factor for *T. gondii* seropositivity and produced some evidence suggesting that individuals living in a greener residential environment were more likely to acquire infection through contacts with local soil contaminated with environmental oocysts of this parasite. The association between contacts with soil and *T. gondii* infections is corroborated by previous research [15, 16, 18]. A study in France also demonstrated that urban and rural residents acquired *T. gondii* infections through different pathways: parasites isolated from urban residents lacked geographic genetic structure, suggesting foodborne infections via products transported over long distances; in contrast, parasites isolated from rural residents exhibited a spatial genetic structure, suggesting a greater importance of local sources of infection [38].

The observed 8.3% seroprevalence of *T. gondii* in this study involving adults was lower than the 13.2% national estimate of unadjusted seroprevalence in children (>5 years of age) and adults in the 2009–2010 NHANES study [8]. The present study was conducted in an urban area with a relatively high educational and socioeconomic status compared to the general US population, which may explain the relatively low observed seroprevalence rate. Local conditions affecting the spread of *T. gondii* might also differ from those in other US regions. Recruitment methods for this study attempted to oversample cat or dog owners. Cat ownership was a risk factor for *T. gondii* seropositivity; this finding was consistent with results of previously conducted studies [10, 11, 14]. Having a dog was a negative predictor of cat ownership; it also tended to be inversely associated with *T. gondii* seropositivity, although the effect was not statistically significant. This finding is consistent with previous research which demonstrated a significant protective effect of dog ownership on *T. gondii* infections [39]. Thus, the effects of oversampling cat and dog owners on *T. gondii* seropositivity in the study population could cancel out each other. Although the seroprevalence estimate from this study is not generalizable due to non-random sampling, findings about risk factors for *T. gondii* infections are likely to reflect transmission pathways of this parasite in central North Carolina.

Furthermore, the present study showed that latent *T. gondii* infections were associated with a detrimental systemic effect reflected in elevated AL, a composite measure of physiologic dysregulation based on multiple biomarkers. Our previous study in the same group of adult residents of North Carolina demonstrated that greener residential environment was associated with reduced AL [32]. Thus, *T. gondii* infections acquired through more frequent contacts with contaminated soils in greener neighborhoods where cats were allowed to roam outdoors could partially offset health benefits of contacts with the natural living environment. This finding shows the importance of minimizing risks of zoonotic infections in green areas.

A limitation of this cross-sectional observational study is that it could only demonstrate statistical associations; it was not designed to establish a cause-effect relationship. While one of the hypotheses of this study was that latent *T. gondii* infections cause chronic inflammation resulting in greater physiological dysregulation and elevated AL measures, there may be alternative explanations for the observed effect. It is possible that individuals with higher AL were more susceptible to *T. gondii* infections (reverse causation). It is also possible that the observed association was due to confounding by unknown behavioral factors that affect the risk of *T. gondii* infection as well as AL.

The beneficial effect of residential vegetation on AL demonstrated in our previous study in the same population [32] remained highly significant after adjusting for *T. gondii* serostatus in the present study. Further stratified analysis showed that the beneficial effects of vegetated land cover were pronounced in both seropositive and seronegative individuals. In seropositive individuals, the detrimental effect of *T. gondii* and the beneficial effect of an IQR increase in residential greenery were of similar magnitudes. However, due to the small sample size (only 15 seropositive individuals with AL data), these effect estimates should be interpreted with caution.

Analysis of associations with individual biomarkers, which comprised the AL index, showed that *T. gondii* infection was linked with a significantly increased aOR of having serum MPO above the 90th percentile and, in analysis of continuous biomarker data, with a higher median concentration of MPO. These findings are logical as MPO is an enzyme involved in immune response to pathogens. Elevated levels of MPO have been linked with inflammation and with cardiovascular diseases [40, 41]. *T. gondii* seropositivity was also linked with elevated levels of VCAM-1 and IL-6 (*p* < 0.05 for each association), although these effects did not remain significant after applying Bonferroni correction for multiple comparisons. Previous research demonstrated associations between *T. gondii* infection and an elevated serum level of VCAM-1 and some pro-inflammatory cytokines [31] corroborating the results of this study. Experimental research also demonstrated that pro-inflammatory cytokines including IL-6 and TNF-α play critical roles in inhibiting the replication of this parasite in humans [42]. Previous studies have also shown associations between *T. gondii* and elevated serum ICAM-1 [43], and reduced serum DHEA (both associations suggesting detrimental health effects) [44]. Those findings were not replicated in the present study possibly due to the small sample size or particular characteristics of the study population.

## Conclusions

The results of this study suggest that handling soil with bare hands, having an outdoor cat and having more greenery around residence were risk factors for *T. gondii* infection in residents of central North Carolina. In turn, *T. gondii* IgG seropositivity was associated with an increased AL suggesting that latent infections have subtle detrimental effects leading to physiological dysregulation and potentially enhancing risk of overt diseases. In contrast, having more greenery around residence was associated with reduced AL in both seronegative and seropositive individuals. The zoonotic *T. gondii* infections may partially offset health benefits of green spaces in a subset of the population.

## Abbreviations

AL: Allostatic load
aOR: adjusted odds ratio
CRP: C-reactive protein
DHEA: Dehydroepiandrosterone
ICAM-1: Intercellular adhesion molecule 1
Ig: Immunoglobulin
IL: Interleukin
IQR: Interquartile range
MPO: Myeloperoxidase
SAA: Serum amyloid A
TNF-α: Tumor necrosis factor α
US EPA: United States Environmental Protection Agency
VCAM-1: Vascular cell adhesion molecule 1
